# Clinical trials and outcome reporting in congenital diaphragmatic hernia overlook long‐term health and functional outcomes—A plea for core outcomes

**DOI:** 10.1111/apa.16409

**Published:** 2022-06-14

**Authors:** Leonie Lewis, Ian Sinha, Paul D. Losty

**Affiliations:** ^1^ Institute of Life Course and Medical Sciences University of Liverpool Liverpool UK; ^2^ Department of Paediatric Respiratory Medicine Alder Hey Children's Hospital NHS Foundation Trust Liverpool UK

## Abstract

**Aim:**

To review the selection, measurement and reporting of outcomes in studies of interventions in Congenital Diaphragmatic Hernia (CDH).

**Methods:**

We searched the Cochrane Central Register of Controlled Trials from 2000–2020 for randomised trials and observational studies. Outcomes reported were classified into seven key domains modelled on the patient journey.

**Results:**

Our search yielded 118 papers; 27 were eligible. The most frequent domains measured were ‘short‐term markers of disease activity’ (17/27), whereas long‐term outcomes (3/27) and outcomes relating to functional health status (8/27) were reported infrequently. There was heterogeneity in the methods and timing of outcome reporting. Primary outcomes were varied and not always clearly stated.

**Conclusion:**

Long‐term health and functional outcomes involving interventional studies in CDH are infrequently reported, which hinders the process of shared decision‐making and evidence‐based healthcare. A CDH core outcome set is needed to standardise outcome reporting that is relevant to both families and healthcare teams.

**Key notes**

- It is crucial that we optimise the utility of clinical trials in Congenital Diaphragmatic Hernia (CDH), but no consensus exists on which parameters should best be measured.
- In trials for CDH, there is heterogeneity in outcome reporting, and long‐term, including functional outcomes, are infrequently reported.
- There is great need for international consensus on CDH outcome reporting.

## 1 INTRODUCTION

Congenital diaphragmatic hernia is a rare disease occurring in 1 in 3000 births^1^ with current mortality now approaching 30%–50%.^2^, ^3^, ^4^ In newborns requiring extracorporeal membrane oxygenation (ECMO) support, a higher mortality of 60% is further evident. CDH babies that survive to hospital discharge may be left with complex long‐term health problems across multiple body systems. Primarily, failure of diaphragmatic closure in utero leads to herniation of abdominal contents into the thoracic cavity causing lung hypoplasia and pulmonary hypertension. Postnatal lung injury occurring in CDH survivors is notable and secondary to aggressive mechanical ventilation. It is estimated that the prevalence of chronic lung disease (CLD) may affect up to 50% of all CDH patients.^5^ Neurological complications, such as motor and cognitive defects and gastrointestinal morbidity including severe gastroesophageal reflux (GERD) are also noteworthy.^5^, ^6^

There are varied post‐natal management strategies that aim to improve both short‐ and long‐term outcomes of infants born with CDH. These include ‘gentle’ ventilation with delayed surgery, as well as the use of ECMO, nitric oxide, and sildenafil.^3^ Despite available post‐natal therapies, there is currently no internationally agreed consensus as to which ‘best outcomes’ should be measured in studies evaluating such interventions in CDH. Outcomes may be selected on the basis of, for example, their financial costs or time constraints, rather than those which would be most informative for healthcare teams and parents. The risk of non‐uniformity amongst clinical trials, and selective outcome reporting on the basis of positive results, can lead to an incomplete and biased representation of ‘best evidence’.^7^, ^8^ Valid comparisons between published studies and synthesis of their results in meta‐analyses are therefore limited by non‐uniform measurement and reporting.

The development of a Core Outcome Set (COS), a standardised set of outcomes, would provide a robust consensus for healthcare professionals regarding CDH.^9^ The aim of this study was to analyse outcome reporting in CDH studies, with a view to designing and developing a valid COS for future research work and wider network collaboration.

### 1.1 Aims

1. To review studies examining post‐natal interventions in CDH to determine which outcomes are measured, and if there are any ‘gaps’ in outcome reporting, or non‐uniformity between studies.
2. To examine trends (if any) in outcome reporting between 2000 and 2020.
3. To determine any associations between outcome reporting and study quality, study type, or patient age group.

## 2 METHODS

The study was performed in accordance with the Preferred Reporting Items for Systematic Reviews and Meta‐Analyses (PRISMA) guidance.^10^ A protocol was developed which defined (I) study objectives, (II) selection criteria, (III) assessment of study quality, (IV) data extraction, and (V) analysis.

### 2.1 Search strategy

We searched Cochrane Central Register of Controlled Trials (CENTRAL) from January 2000 to December 2020, using the pre‐determined heading term ‘Congenital Diaphragmatic Hernia’. CENTRAL is a comprehensive database of clinical trials and encompasses trials from PubMed, Embase, CINAHL, ClinicalTrials.gov and WHO ICTRP. The database was last searched on 25 August 2021.

The study authors screened all potential studies based on title and abstract. The selected studies were then read in full to screen for eligibility.

Studies included were (i) published randomised controlled trials (RCTs) and (ii) observational studies of any post‐natal care interventions in CDH. There were no limitations set on the age of study participants.

Studies excluded were duplicates, abstract‐only papers, manuscripts published before 2000, and those not in the English language. Studies of pre‐natal interventions for CDH (including fetoscopic endoluminal tracheal occlusion ‘FETO’ and cord clamping) and animal studies were also excluded.

### 2.2 Data extraction and quality assessment

Data from selected eligible studies were extracted by two authors (LL and IS). Extracted data included study characteristics with their main findings and results.

Study characteristics included (a) study design, (b) single or multi‐centre study, (c) number of patients, (d) age of patients, and (e) intervention(s).

Study results consisted of (i) outcomes reported, (ii) primary outcome reporting, and whether the study focused on (iii) long or short‐term outcomes. Outcomes were then grouped into categorical domains.

The study authors assessed observational study quality using the methodological index for non‐randomised studies (MINORS).^11^ Study quality for RCTs was assessed using the Cochrane risk of bias tool for randomised trials.^12^

### 2.3 Data analysis

**FIGURE 1:**
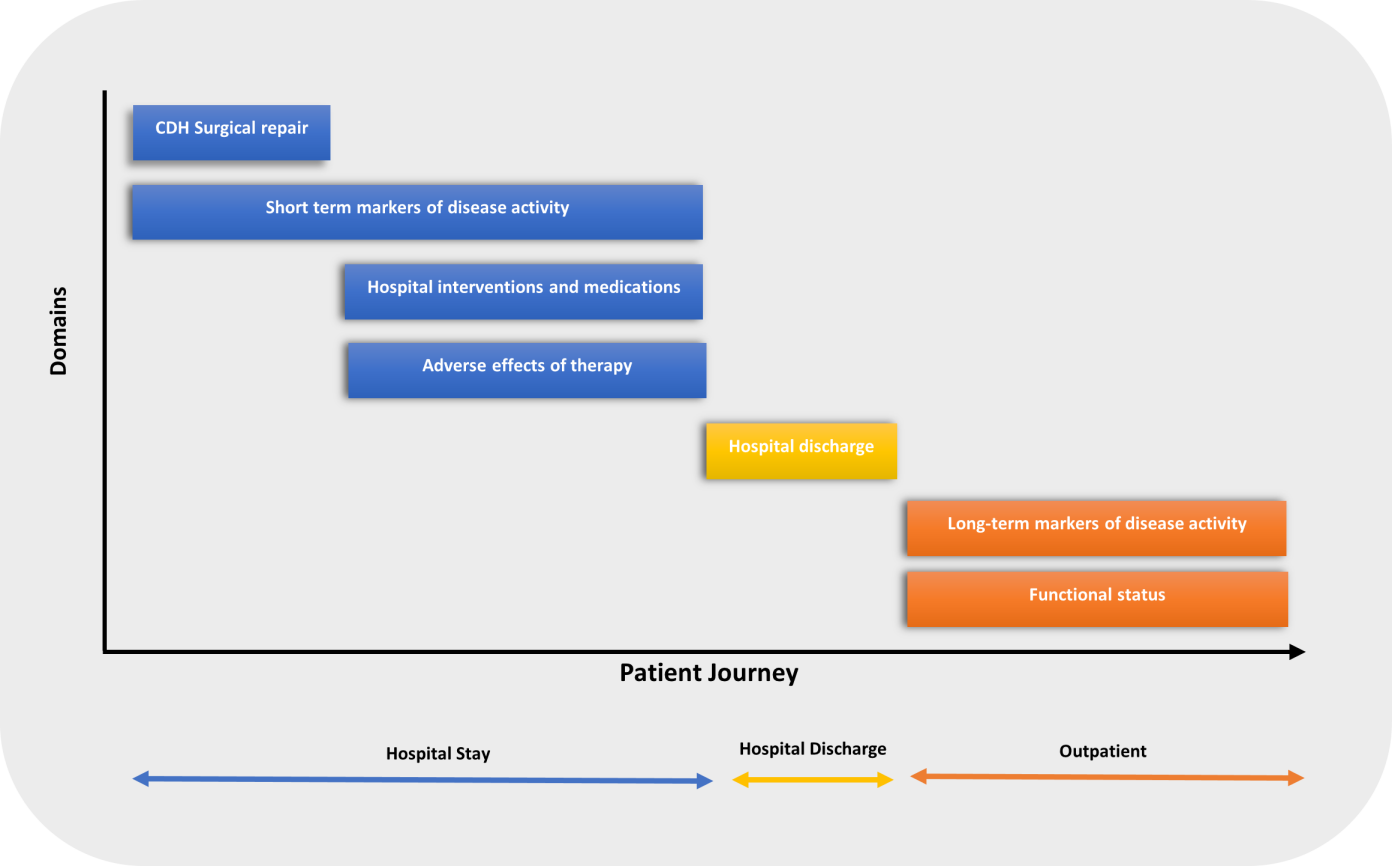
Seven key domains modelled on the patient journey

Outcomes from each study were extracted and then categorised by open discussion between the study investigators into seven predetermined domains. (i) CDH surgical repair; (ii) short‐term markers of disease activity; (iii) hospital interventions and medications; (iv) adverse effects of therapy; (v) hospital discharge; (vi) long‐term markers of disease activity; and (vii) functional health status. Domains were based on Sinha et al.'s publication reporting on outcomes in paediatric asthma^9^ and modelled on the CDH patient journey throughout their hospital stay and then post hospital discharge (Figure 1).

Outcomes were also classified as short term (measured <1 year) or long term (measured >1 year).

Study interventions were categorised into (a) use of ECMO, (b) cardiopulmonary drugs, (c) anti‐reflux medications, (d) neurocognitive training, (e) inspiratory muscle training, (f) ventilation strategies, (g) surgical CDH repair, (h) surgical CDH repair and mode strategy of ventilation. Occasionally, a study intervention was not explicitly listed by authors and was therefore defined as ‘unclear’.

To determine how trends in outcome reporting changed over time, studies were also classified by year of publication.

To determine outcome(s) reporting by age category, each study was classified by the age range of CDH patients. Categories were newborns (<28 days), infants (<1 year), children and adolescents (<18 years), and adults (>18 years).

## 3 RESULTS

### 3.1 Study search and selection

**FIGURE 2:**
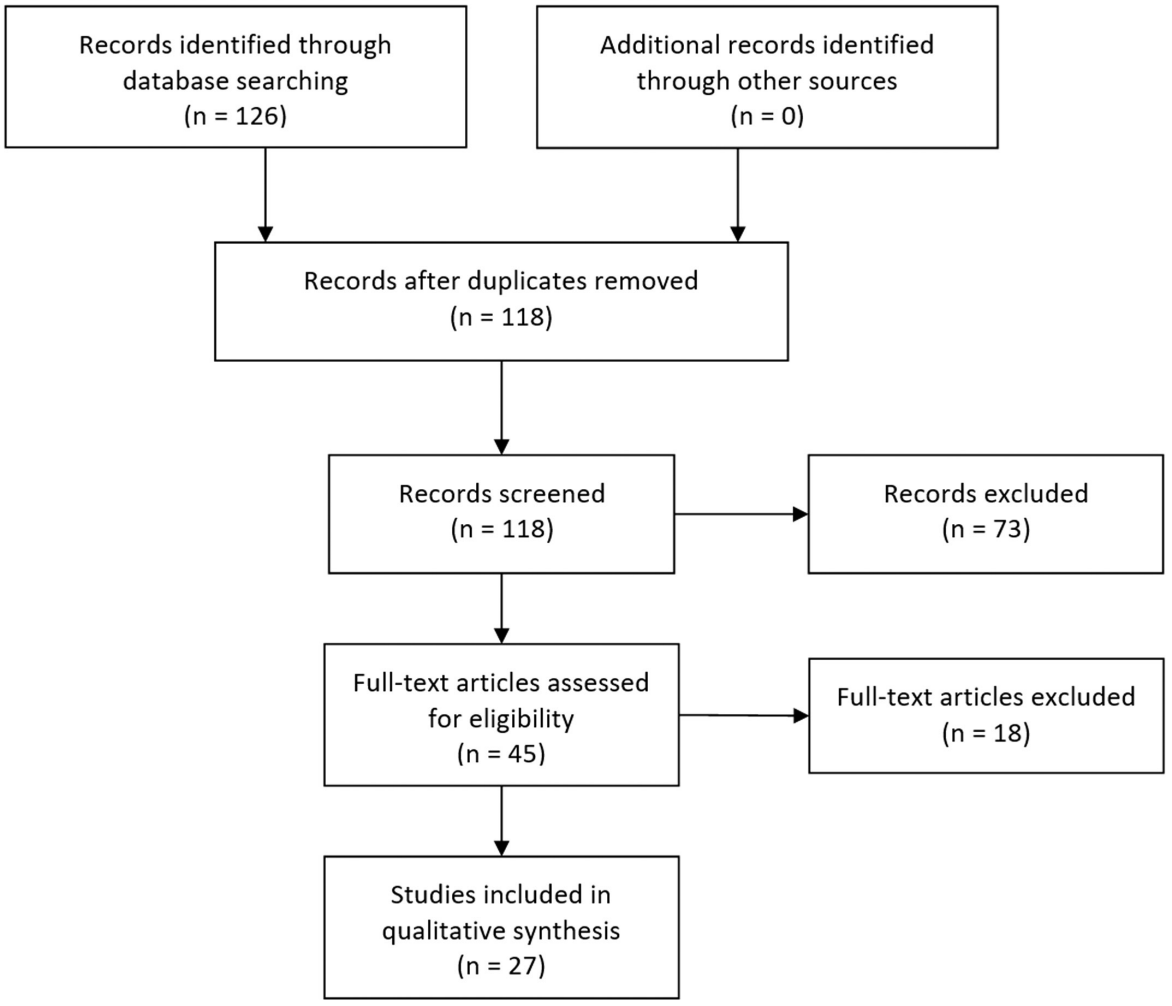
PRISMA flowchart

The search of CENTRAL yielded 126 papers, and after removal of 8 duplicates, 118 papers were screened. Titles and abstracts were then assessed for full eligibility, excluding 73 papers. The remaining 45 publications were read in full, and a further 18 papers excluded. Excluded studies were duplicates, abstract‐only papers, manuscripts published before year 2000, those not in the English language, animal studies, and those describing pre‐natal CDH interventions. We included 27 studies^13^, ^14^, ^15^, ^16^, ^17^, ^18^, ^19^, ^20^, ^21^, ^22^, ^23^, ^24^, ^25^, ^26^, ^27^, ^28^, ^29^, ^30^, ^31^, ^32^, ^33^, ^34^, ^35^, ^36^, ^37^, ^38^, ^39^ of which 13 were RCTs^13^, ^14^, ^15^, ^16^, ^17^, ^18^, ^19^, ^20^, ^21^, ^22^, ^23^, ^24^, ^25^ and 14 articles were observational studies.^26^, ^27^, ^28^, ^29^, ^30^, ^31^, ^32^, ^33^, ^34^, ^35^, ^36^, ^37^, ^38^, ^39^ Figure 2 shows the PRISMA flowchart for the study review.

### 3.2 Study characteristics

Study characteristics are summarised in Table S1. Overall, the studies included 2596 CDH patients. The mean number of index CDH patients was 96 per publication (range 5–691); 13/27 studies were RCTs,^13^, ^14^, ^15^, ^16^, ^17^, ^18^, ^19^, ^20^, ^21^, ^22^, ^23^, ^24^, ^25^ and 14/27 were observational studies^26^, ^27^, ^28^, ^29^, ^30^, ^31^, ^32^, ^33^, ^34^, ^35^, ^36^, ^37^, ^38^, ^39^; 7/27 studies were multicentre collaborative works. Studies emerged from a variety of countries worldwide including the UK, Netherlands, Belgium, USA, Canada, Japan, Egypt, and Australia.

Seventeen of 27 studies (63%) included CDH newborns, 4/27 (15%) infants,^13^, ^26^, ^32^, ^35^ 4/27 (15%) children and adolescents <18 years^18^, ^22^, ^33^, ^39^ and 2/27 (7%) publications included children, adolescents, and adults with CDH.^27^, ^37^ No studies included exclusively adults with CDH.

Interventions described amongst these many studies were wide‐ranging and classified into 10 categories, with studies relating to surgical CDH repair, ventilation, and cardiopulmonary drugs being the most common.

Sixteen of 27 (59%) CDH studies reported only short‐term outcomes (<1 year); 6/27 (22%) reported only long‐term outcomes (>1 year); and 5/27 (19%) scrutinised both short‐ and long‐term outcomes.

### 3.3 Study quality

Table S2 shows the Cochrane ‘risk of bias’ for the randomised trials included in this study.^12^ Overall risk of bias was rated as ‘low risk’ if all domains fell under this category, ‘some concern’ if the trial had one domain in this category, and ‘high risk’ if more than one domain showed ‘some concern’ or at least one category domain was ‘high risk’. Seven papers showed ‘some concern’ for risk of bias, and six papers showed a ‘high risk’ of bias. No papers showed a ‘low risk’ of bias.

Table S3 shows the observational studies as rated by the methodological index for non‐randomised studies (MINORS).^11^ Non‐comparative studies were given an overall score out of 16, and comparative studies a score out of 24. Overall scores ranged from 54%–81%.

### 3.4 Study results

#### 3.4.1 Domains and outcomes measured

**FIGURE 3:**
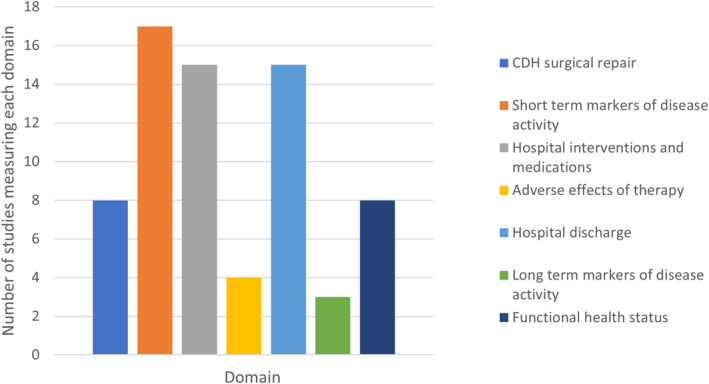
Domain popularity

Table 1 shows a breakdown of which outcomes are included in each domain, as well as outcome and domain frequency across all studies. Short‐term markers of disease activity was the most frequently reported domain, 17/27 (63%) of papers, followed by hospital interventions and medications (15/27, 56%), hospital discharge (15/27, 56%), surgical CDH repair (8/27, 30%), functional status (8/27, 30%), adverse effects of therapy (4/27, 15%), and long‐term markers of disease activity (3/27, 11%). These findings are depicted in Figure 3.

**TABLE 1:** Frequency with which outcomes were reported in published studies

Domain	Subdomain	Outcome	Number of studies which measured outcome *n* (%)
CDH surgical repair (*n* = 8, 29.6%)		Timing of repair	4 (14.8)
		Primary or Patch repair	1 (3.7)
		Ease of intubation	1 (3.7)
		% CO_2_ exhaled during operation	1 (3.7)
		Intraoperative or postop complications	2 (7.4)
		Conversion to open surgery	1 (3.7)
		Hernia recurrence	2 (7.4)
Short‐term markers of disease activity (*n* = 17, 62.9%)	General markers	Medical history and examination	5 (18.5)
		Vital signs	7 (25.9)
	Respiratory markers	Oxygenation Index (OI)	3 (11.1)
		Evidence of pulmonary hypertension	5 (18.5)
		Lung function testing	1 (3.7)
		CXR	3 (11.1)
		Pulmonary hypoplasia at post‐mortem	1 (3.7)
	Neurological markers	Neurological scan – USS or NIRS	1 (3.7)
	Gastrointestinal markers	Evidence of gastro‐oesophageal reflux disease/pH monitoring	3 (11.1)
	Laboratory markers	Blood gases	8 (29.6)
		Brain Natriuretic Peptide (BNP)	2 (7.4)
Hospital interventions and medications (*n* = 15, 55.5%)	Interventions	ECMO	8 (29.6)
		Ventilation	10 (37.0)
		Oxygen	5 (18.5)
		Chest tube	1 (3.7)
		Type of feeding e.g. NG or Gastrostomy tube	2 (7.4)
	Medications	Pulmonary or cardiac drugs	6 (22.2)
		Surfactant	3 (11.1)
		Anti‐reflux agents	1 (3.7)
		Analgesia	2 (7.4)
	Other	Financial cost of treatment	1 (3.7)
		Intervention ‘free’ days	1 (3.7)
Adverse effects of therapy (*n* = 4, 14.8%)		Treatment failure	1 (3.7)
		Haematological complications	2 (7.4)
		Renal complications	2 (7.4)
		Central line sepsis	1 (3.7)
		Pneumothorax	1 (3.7)
		Electrolyte abnormalities	1 (3.7)
		Dose of intervention therapy	1 (3.7)
Hospital discharge (*n* = 15, 55.5%)		Mortality rate	13 (48.1)
		Age at death	3 (11.1)
		Hospital discharge rate	2 (7.4)
		Duration of hospital stay/age at discharge	3 (11.1)
		Discharged with treatment/medications	2 (7.4)
Long‐term markers of disease activity (>1 year) (*n* = 3, 11.1%)		History and Clinical examination	2 (7.4)
		Medications	1 (3.7)
		Echocardiogram	1 (3.7)
		Pulmonary function testing and cardiopulmonary exercise testing (CPET)	3 (11.1)
Functional health status (*n* = 8, 29.6%)		Use of a speciality medical clinic	2 (7.4)
		Neurological function	3 (11.1)
		Occupational or speech therapy	1 (3.7)
		Social worker	1 (3.7)
		Education level/school function	2 (7.4)
		Socioeconomic status	2 (7.4)
		Behaviour and attention	2 (7.4)
		Self‐esteem	2 (7.4)
		Opinion of physical fitness and activity levels	2 (7.4)
		QoL – child or carer	3 (11.1)

The average number of outcomes measured by each published study was 6. The most popular outcome reported was mortality rate (%) measured in 13/27 (48%) of studies. This was followed by use of ventilation mode (10/27, 37% of studies), ECMO (8/27 29% of studies), and blood gas analyses (8/27, 29% of studies).

#### 3.4.2 Primary outcomes in RCTs

A primary outcome was specified in 10/13 (77%) of RCTs. The remaining 3 (23%) of RCTs reported multiple outcomes but did not clearly specify which was their primary outcome. When a primary outcome was reported by published studies, these fell into domains relating to CDH surgical repair (intubating status as measured by the Copenhagen scale – time taken to intubate and the number of attempts), short‐term markers of disease activity (arterial CO_2_ level, and pH monitoring), hospital discharge (mortality/survival rate), long‐term markers of disease activity (cardiopulmonary exercise training), and functional status (neurological function). ‘Hospital discharge’ was the most popular domain for primary outcome. In 5/13 RCTs, the primary outcome fell under this domain.

#### 3.4.3 Trends in outcome reporting based on study quality

There was little difference observed in outcomes reported between high and lower quality RCTs (as measured by the Cochrane risk of bias for randomised trials^12^).

#### 3.4.4 Trends in CDH outcome reporting during 2000–2020

Due to the small numbers of papers included here, we could not draw any firm or valid conclusions on the trends in outcome reporting over time.

#### 3.4.5 Trends in outcome reporting by age category and study type

As the age of CDH patients increased, short‐term outcomes decreased in popularity and long‐term outcomes thereafter increased.

There was some difference in domain popularity amongst the RCTs and observational CDH studies; the greatest disparity here was for ‘hospital interventions and medications’ followed by ‘short‐term markers of disease activity’ which were both more equally popular amongst randomised studies.

## 4 DISCUSSION

Our primary aim was to review studies examining post‐natal interventions in CDH to see which outcomes are measured, and if there are any visible gaps in outcome reporting, or non‐uniformity between published studies. The second aim was to examine trends (if any) over time. The third aim was to determine any associations between outcome reporting with regard to study quality, study type, or patient age group.

In RCTs and observational studies, short‐term severity of CDH and outcomes related to hospital discharge were the most frequently reported outcome domains. There was wide variability in the choice of outcome selected and reported. Only 77% (10/13) of RCTs measured a primary outcome and again there was variability in the choice of outcome. The other 23% (3/13) of RCTs reported multiple outcomes but did not specify which was considered their primary study outcome. The primary outcome is considered a metric of greatest importance to healthcare outcomes research. Sample size calculations are usually performed using the primary outcome and this then reduces the risk of false‐negative findings. Having several primary outcomes can be problematic as this risks false‐positive errors from statistical testing of too many outcomes so is therefore not recommended.^40^

Markers of functional status, including health‐related quality of life (QoL) and education, were rarely measured.

These findings are particularly noteworthy and have also been noted in other childhood illnesses such as those examining paediatric asthma.^9^ Functional outcomes are crucially important in the day‐to‐day lives of CDH survivors. The lack of functional outcome reporting highlights the requirement for strong networking with CDH patients and families.

Long‐term outcomes were likewise less frequently reported. When reported, we noted that they were often ‘one‐off measurements’ rather than a set of clear outcomes addressing long‐term health. For example, in one trial which measured lung spirometry and cardiopulmonary exercise testing in those aged 5–20 years, CDH patients were only tested on one single occasion.^27^ These observations thus highlight the absolute necessity to develop robust policies on CDH follow‐up which has been suggested before.^41^, ^42^, ^43^ Ijsselstijn et al.^44^ have recently proposed a follow‐up programme for patients born with congenital anomalies throughout their childhood and into early adulthood.

In this study, we further documented that CDH health outcomes were often measured by different methods and at different time points. For example, pulmonary hypertension was diagnosed in three different ways by (a) echocardiography, (b) electrocardiogram (ECG), and (c) clinical examination. Varied reporting methods here likely mean that these individual publications cannot be adequately compared and that their usefulness is therefore somewhat limited. Lally et al.^2^ and the CDH Study Group published a consensus on standardised reporting in CDH, stating that ‘standardizing reporting is imperative in determining optimal outcomes’.

We have shown with the heterogeneity observed between published study outcomes, a clear need for patients with CDH to have a well‐defined COS. Core outcome sets have been developed in paediatric asthma,^45^ neonatology,^46^ and prenatal foetal interventions in CDH.^47^ We are currently planning to work with CDH UK and the COMET initiative to develop a bespoke core outcome set for postnatal interventions in CDH. This will require the active participation and engagement of all stakeholder groups, notably healthcare professional experts, clinicians, researchers, CDH patients, and families. Other groups to support this collaborative work plan would include Congenital Diaphragmatic Hernia International (CDHi)^48^ and the CDH EURO Consortium—a network of expert health professionals set up to standardise CDH research and which has undertaken multicentre trials such as the ‘VICI’ ventilation trial.^49^

To the best of our knowledge this is the first study to comprehensively investigate outcome reporting in studies of post‐natal interventions in CDH.

Although it is acknowledged that we did not formally investigate selective reporting bias, this could have had important implications. When scrutinising publications and comparing the outcomes that were measured to those reported, we did uncover some evidence of selective reporting bias. For example, various RCTs did not report measurements on vital clinical signs despite specifying these as an outcome measure.^16^, ^25^

The main limitation of the current study was that analysis was somewhat hindered by the relatively small number of eligible CDH studies with few participants. As study authors, we believe that this is most likely linked to the rarity of the disease and possibly the lack of super‐centralisation of care. Centralisation of care may allow ‘high‐volume’ centres to become much more specialised in treating CDH, thereby facilitating ‘better robust network trials’ involving larger numbers of eligible CDH patients.

## 5 CONCLUSION

This current study demonstrates wide heterogeneity in outcome reporting in CDH trials, meaning comparisons are very limited. We also note a significant lack of reporting of long‐term outcomes including health‐related quality of life (QoL). This study crucially highlights the importance of international consensus on outcome reporting, particularly those linked to long‐term follow‐up. We plan to work actively with healthcare professional experts, CDH UK, the COMET initiative, and other key stakeholder groups to develop a robust COS for post‐natal interventions in CDH.

## CONFLICT OF INTEREST

The authors have no competing interests to declare.

## Supporting information


Table S1

Table S2

Table S3
