# Supplementary material for: Clinical trials and outcome reporting in congenital diaphragmatic hernia overlook long‐term health and functional outcomes—A plea for core outcomes
Source: Acta Paediatr. 2022 Jun 14;111(8):1481–9. doi: 10.1111/apa.16409 (PMC9542300; doi:10.1111/apa.16409)
Supplement: Supplementary file 1 — Table S1 [file APA-111-1481-s001.docx]

**Supplementary Table S1: Study Characteristics**

| **Study characteristic** | **Category** | **Number of studies** | **Percentage of studies (%)** |
| --- | --- | --- | --- |
| Year of publication | 2000-2004 | 8 | 30 |
|  | 2005-2009 | 1 | 4 |
|  | 2010-2014 | 2 | 7 |
|  | 2015-2019 | 13 | 48 |
|  | 2020 | 3 | 11 |
| Study type | Randomised controlled trials | 13 | 48 |
|  | Observational studies | 14 | 52 |
| No. of centres | Single Centre | 20 | 74 |
|  | Multi Centre | 7 | 26 |
| No. of CDH patients | <25 | 8 | 30 |
|  | 25-49 | 8 | 30 |
|  | 50-99 | 4 | 15 |
|  | 100-299 | 4 | 15 |
|  | 300-700 | 3 | 11 |
| Age of patients | Newborns (<28 days) only | 17 | 63 |
|  | Infants (<1 year) only | 4 | 15 |
|  | Children and adolescents (<18 years) only | 4 | 15 |
|  | Children and adolescents (<18 years), and Adults (>18 years) | 2 | 7 |
|  | Adults (>18 years) only | 0 | 0 |
| Intervention | Neuro-cognitive training | 1 | 4 |
|  | Inspiratory muscle training | 1 | 4 |
|  | ECMO | 1 | 4 |
|  | Cardiopulmonary drugs (iNO, Milrinone, Treprostinil, Sildenafil) | 4 | 15 |
|  | Anti-reflux drugs | 1 | 4 |
|  | Patient position (prone or supine) | 1 | 4 |
|  | Ventilation | 6 | 22 |
|  | Surgical CDH repair | 8 | 30 |
|  | Surgical CDH repair and ventilation | 2 | 7 |
|  | None/unclear | 2 | 7 |
| Outcomes | Short-term (<1 year) | 16 | 59 |
|  | Long-term (>1 year) | 6 | 22 |
|  | Both | 5 | 19 |
