# Supplementary material for: Clinical trials and outcome reporting in congenital diaphragmatic hernia overlook long‐term health and functional outcomes—A plea for core outcomes
Source: Acta Paediatr. 2022 Jun 14;111(8):1481–9. doi: 10.1111/apa.16409 (PMC9542300; doi:10.1111/apa.16409)
Supplement: Supplementary file 2 — Table S2 [file APA-111-1481-s003.docx]

**Supplementary Table S2: Cochrane Risk of Bias For Randomised Trials (n=13)**

| Primary author | Domain 1: Risk of bias arising from the randomisation process | Domain 2: Risk of bias due to deviations from the intended interventions | Domain 3: Risk of bias due to missing outcome data | Domain 4: Risk of bias in measurement of the outcome | Domain 5: Risk of bias in selection of the reported result | Overall risk of bias |
| --- | --- | --- | --- | --- | --- | --- |
| Bestebreurtje^13^ | Low risk | Some concerns | Low risk | Low risk | Low risk | Some concerns |
| Bishay^14^ | Low risk | Low risk | Low risk | Low risk | High risk | High risk |
| Guevorkian^15^ | Some concerns | Some concerns | Low risk | Low risk | Low risk | High risk |
| Hirschl^16^ | Low risk | Some concerns | Low risk | Low risk | Low risk | Some concerns |
| Jacobs^17^ | Low risk | Some concerns | Low risk | High risk | Low risk | High risk |
| Moawd^18^ | Low risk | Some concerns | Low risk | Low risk | Low risk | Some concerns |
| Moustafa^19^ | Low risk | Some concerns | Low risk | Low risk | Low risk | Some concerns |
| Mychaliska^20^ | Low risk | Some concerns | Low risk | Low risk | Low risk | Some concerns |
| NINOS^21^ | Low risk | Some Concerns | Some concerns | Low risk | Low risk | High risk |
| Schiller^22^ | Low risk | Some Concerns | Low risk | High risk | Low risk | High risk |
| Snoek^23^ | Low risk | Some concerns | Low risk | Low risk | Low risk | Some concerns |
| Snoek^24^ | Low risk | Some concerns | Low risk | Low risk | Low risk | Some concerns |
| Wu^25^ | some concerns | Some concerns | Some concerns | Low risk | Low risk | High risk |
