# Supplementary material for: Clinical trials and outcome reporting in congenital diaphragmatic hernia overlook long‐term health and functional outcomes—A plea for core outcomes
Source: Acta Paediatr. 2022 Jun 14;111(8):1481–9. doi: 10.1111/apa.16409 (PMC9542300; doi:10.1111/apa.16409)
Supplement: Supplementary file 3 — Table S3 [file APA-111-1481-s002.docx]

**Supplementary Table S3: Methodological index for non-randomised studies (MINORS) score (n=14)**

| Primary author | 1. Clearly stated aim | 2. Inclusion of consecutive patients | 3. prospective collection of data | 4. Endpoints appropriate to the aim of the study | 5. Unbiased assessment of study endpoint | 6. Follow up period appropriate | 7. Loss to follow up less than 5% | 8. Prospective calculation of study size | 9. Adequate control group | 10. Contemporary groups (no historical comparison) | 11. baseline equivalence of groups | 12. adequate statistical analysis | Overall Score |
| --- | --- | --- | --- | --- | --- | --- | --- | --- | --- | --- | --- | --- | --- |
| Bevilacqua^26^ | **2** | **1** | **2** | **2** | **0** | **2** | **1** | **0** | **N/A** | **N/A** | **N/A** | **N/A** | **10/16 (63%)** |
| Bojanic^27^ | **2** | **2** | **0** | **2** | **0** | **1** | **1** | **0** | **2** | **2** | **2** | **0** | **14/24 (58%)** |
| Boloker^28^ | **2** | **2** | **1** | **2** | **2** | **2** | **2** | **0** | **N/A** | **N/A** | **N/A** | **N/A** | **13/16 (81%)** |
| Chamond^29^ | **2** | **2** | **1** | **2** | **0** | **2** | **1** | **0** | **2** | **2** | **1** | **0** | **15/24 (63%)** |
| Cruz^30^ | **2** | **2** | **2** | **2** | **2** | **2** | **2** | **0** | **2** | **2** | **1** | **0** | **19/24 (79%)** |
| Desfrere^31^ | **2** | **2** | **2** | **2** | **0** | **2** | **2** | **0** | **2** | **2** | **2** | **0** | **18/24 (75%)** |
| Harting^32^ | **2** | **2** | **2** | **2** | **0** | **2** | **2** | **0** | **2** | **2** | **1** | **2** | **18/24 (75%)** |
| Kubota^33^ | **2** | **1** | **2** | **2** | **1** | **2** | **1** | **0** | **2** | **2** | **0** | **0** | **15/24 (63%)** |
| Lally^34^ | **2** | **1** | **2** | **2** | **0** | **2** | **1** | **0** | **N/A** | **N/A** | **N/A** | **N/A** | **10/16 (63%)** |
| Lawrence^35^ | **2** | **2** | **1** | **2** | **2** | **2** | **2** | **0** | **2** | **2** | **1** | **0** | **18/24 (75%)** |
| Maier^36^ | **2** | **1** | **2** | **2** | **2** | **2** | **1** | **1** | **2** | **2** | **2** | **2** | **21/24 (88%)** |
| Mesas Burgos^37^ | **2** | **1** | **2** | **2** | **0** | **2** | **0** | **0** | **2** | **2** | **0** | **0** | **13/24 (54%)** |
| Okuyama^38^ | **2** | **2** | **0** | **2** | **0** | **2** | **1** | **0** | **2** | **2** | **1** | **0** | **14/24 (58%)** |
| Turchetta^39^ | **2** | **1** | **0** | **2** | **0** | **1** | **2** | **0** | **2** | **2** | **2** | **0** | **14/24 (58%)** |
